# The role of resuscitation promoting factors in pathogenesis and reactivation of *Mycobacterium tuberculosis *during intra-peritoneal infection in mice

**DOI:** 10.1186/1471-2334-7-146

**Published:** 2007-12-17

**Authors:** Sergey Biketov, Vasilii Potapov, Elena Ganina, Katrina Downing, Bavesh D Kana, Arseny Kaprelyants

**Affiliations:** 1 Scientific Research Center for Applied Microbiology and Biotechnology, Moscow Region, Russia; 2 MRC/NHLS/WITS Molecular Mycobacteriology Research Unit, DST/NRF Centre of Excellence for Biomedical TB Research, School of Pathology of the University of the Witwatersrand and the National Health Laboratory Service, Johannesburg, South Africa; 3 Bakh Institute of Biochemistry, Russian Academy of Sciences, Moscow, Russia

## Abstract

**Background:**

*Mycobacterium tuberculosis *can enter into a dormant state which has resulted in one third of the world's population being infected with latent tuberculosis making the study of latency and reactivation of utmost importance. *M. tuberculosis *encodes five resuscitation promoting factors (Rpfs) that bear strong similarity to a lysozyme-like enzyme previously implicated in reactivation of dormant bacteria *in vitro*.

We have developed an intraperitoneal infection model in mice, with immune modulation, that models chronic infection with similar properties in mouse lungs as those observed in the murine aerosol infection model. We have assessed the behavior of mutants that lack two or three *rpf *genes in different combinations in our intraperitoneal model.

**Methods:**

C57Bl/6 mice were intraperitonealy infected with H37Rv wild type *M. tuberculosis *or mutant strains that lacked two or three *rpf *genes in different combinations. After 90 days of infection aminoguanidine (AG) or anti-TNFα antibodies were administrated. Organ bacillary loads were determined at various intervals post infection by plating serial dilutions of organ homogenates and enumerating bacteria.

**Results:**

We found that the *rpf *triple and double mutants tested were attenuated in their ability to disseminate to mouse lungs after intraperitoneal administration and were defective in their ability to re-grow after immunosuppression induced by administration of aminoguanidine and anti-TNFα antibodies.

**Conclusion:**

Rpf proteins may have a significant physiological role for development of chronic TB infection and its reactivation *in vivo*.

## Background

*Mycobacterium tuberculosis *(MTB), the causative agent of tuberculosis (TB) is responsible for the largest number of deaths attributive to a single human pathogen. This exquisitely adapted bacterium has infected almost a third of the world's population [1] with approximately 8 million new cases and several million deaths every year. The majority of infected people carry the tubercle bacillus in a dormant or latent form and hence display no signs of primary disease. However, these people carry a 5–10% life time risk of developing reactivation disease and HIV positive individuals carry a >10% risk of developing tuberculosis (2 – 23% during lifetime in HIV-negative populations and 5 – 10% per year for HIV-infected populations [2]). As a result the study of the clinically latent state and subsequent reactivation has been the subject of intense investigation and is considered as an essential part of the general strategy to prevent the spread of TB. To this end novel specific experimental models should be evaluated and applied in the search and testing of new targets and chemical interventions for the prevention and treatment of dormant forms of TB infection.

Several *in vitro *and *in vivo *animal models have been established in an attempt to mimic the latent state and subsequent reactivation disease. Detailed characterisation of these models in recent years has contributed significantly to our understanding of the biology of MTB [3-5]. Modern molecular technologies (transcriptional profiling using microarrays, proteomic analyses, and real-time quantitative reverse-transcription PCR) have been employed to characterise MTB in the various persistence models currently in use, which have revealed both similarities and differences between them [6,7]. Some *in vitro *models suggest that viable cells of MTB have the capacity to adopt a non-culturable state and that culturability can be restored by providing nutrients in the form of fresh media [4]. This resembles the situation *in vivo*, in the Cornell model, which is probably most adequate, where bacteria are able to assume a dormant state post drug treatment and are then able to reactivate upon generalized immune suppression [8,9] or spontaneously [10]. However, the disadvantages of this model are the long time needed to complete studies and significant variability of the results in different animal populations [11]. The widely used murine model of chronic TB infection is less complicated than the Cornell model but its relevance to paucibacillary tuberculosis latency in humans is questionable. With this model bacteria can be administered either intravenously or aerogenically, with the former the results following infection in mice are highly dependent on the infection dose (which varies significantly in different studies), the way the inoculum is prepared, and the mouse strain. Aerogenic challenge with a low dose of bacteria provides more consistent results [12,13]. This model permits long-term survival of the mice with relatively high and essentially stable numbers of bacteria in the lungs and spleen [14]. Although less frequently used, intra-peritoneal (IP) administration of MTB results in a moderate and stable bacterial load in the organs over a period of 50 weeks post-infection [15].

An understanding of the molecular mechanisms that controls the transition of viable mycobacteria to a dormant state and vice versa will be of great value for the development of novel interesting targets and new compounds that have activity against latent forms of tuberculosis. The resuscitation-promoting factor (Rpf) is a member of a protein family that is found throughout the actinobacteria. In *Micrococcus luteus*, the addition of Rpf (a secreted protein, which is active at picomolar concentrations) was necessary for restoration of culturability from a dormant state, [16] furthermore Rpf also stimulated multiplication of normal viable bacteria [17,18]. Disruption of the *rpf *gene was not possible in *M. luteus *in the absence of a second functional copy, strongly suggesting essentiality of this protein for normal growth [17]. MTB contains five *rpf*-like genes, whose products, RpfA-E, when expressed as recombinant proteins in *E. coli*, have similar properties to that of *M. luteus *Rpf [18]. The presence of *rpf-*like genes in MTB raises the possibility that their products may also control bacterial growth and resuscitation in vivo. Unlike the *M. luteus *Rpf, none of the Rpf-like proteins in MTB are individually essential for growth in vitro or pathogenesis in vivo [19-21]. We have shown that two different triple mutants lacking *rpfA*, *rpfC *and either *rpfD *or *rpfB *were defective for resuscitation *in vitro *however, their growth characteristics *in vitro *were similar to wild type. Furthermore these triple mutants revealed significant differential attenuation in virulence in a mouse model of semi-acute TB infection and showed reduced ability to proliferate in the lungs and spleens of infected mice [20]. Recently it has been reported that deletion of Rv1009 (*RpfB*) results in delayed reactivation of bacterial growth after immune suppression in the mouse model of chronic tuberculosis [22].

In this study we demonstrate that multiple *rpf *deletion mutants revealed differential attenuation in TB chronic model established by the intra-peritoneal route of infection and in reactivation after immunosuppression.

## Methods

### Bacteria, growth conditions and mouse strains

Mutant strains of MTB that lacked two or three of the *rpf *genes are described previously [20]. MTB H37Rv wild type and mutant strains were grown in Middlbrook 7H9 media containing 0.05% Tween 80 and 10% of Growth Supplement ADC (HIMEDIA) to mid-log phase and then stored in aliquots at -70°C until further use.

### Mouse infections

Six to eight week old C57BL/6 female mice (supplied from the Pushino Animal Breeding Facility) were maintained in animal facilities of SRCAMB in Obolensk which is in compliance with the Standards for Humane Care and Use of Laboratory Animals (OLAW Assurance number A5500-01, expires on 10/31/2011). The animals were maintained under Animal Biosafety Level 3 conditions throughout the study in cages with filtered air and on sterile water. The animal protocol employed in this study was approved by the Institutional Animal Care and Use Committee.

Mice were infected intraperitoneally with frozen stocks of wild type and mutant strains, using an infection dose of ca. 10^3 ^colony forming units (CFUs) per mouse in 0.2 ml of suspension. At various times after infection the mice were euthanized with CO_2 _and portions of the lungs and spleen were homogenized in PBS with 0.05% Tween 80. The numbers of viable bacteria in the lungs were monitored over time by plating serial dilutions of organ homogenates from individual mice (6 animals per time point) on to Middlebrook 7H11 (HIMEDIA) agar. For the first time point (10 days) homogenates from different mice were combined before plating to decrease the limit of detection, given the very low numbers of organisms present at this time point. The CFUs were counted after 21 days incubation at 37°C. The data were expressed as the log_10 _value of the mean number of bacteria recovered from different animals.

### Aminoguanide and anti TNFα Antibody administration

Aminoguanidine carbonate (AG) (Sigma) was administered 90 days postinfection (1 ml, 1% wt/vol) with a stomach pump for a period of 14 days. Monoclonal antibodies to TNF-α (kindly provided by Dr. Abbasova S.G.) were administrated intraperitoneally in physiological saline (100 μg in 0.2. ml per mouse per day for 10 days).

### Histopathology

Mice lungs were fixed in 10% formaline, embedded in paraffin, sectioned and stained with hematoxylin/eosin.

## Results

### IP model of chronic TB infection

**Figure 1:**
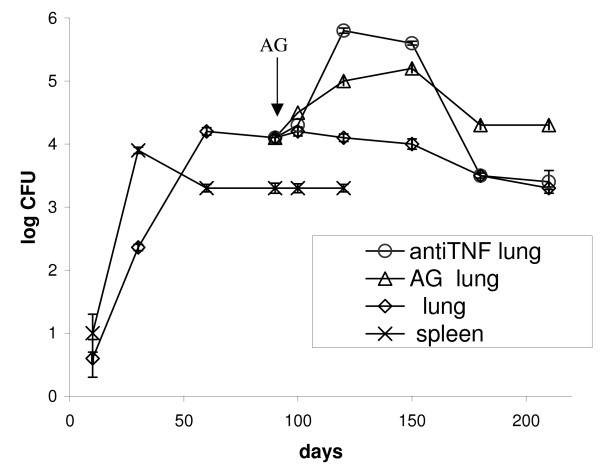
**Growth kinetics of *M. tuberculosis *in organs after intraperitonel infection of C57Bl/6 mice**. C57Bl/6 mice were infected via the intraperitoneal route with 10^3 ^CFUs per animal. After 90 days of infection aminoguanidine (AG) or anti-TNFα antibodies were administered. For details see Materials and Methods. Mice were sacrificed at several time points post infection and the organ bacillary loads were estimated by plating serial dilutions of spleen and lung homogenates (from 6 mice per time point) onto agar medium. Each infected group contained 60 mice. The curves shown represent the average of one experiment and each experiment was repeated at least two times.

**Figure 2:**
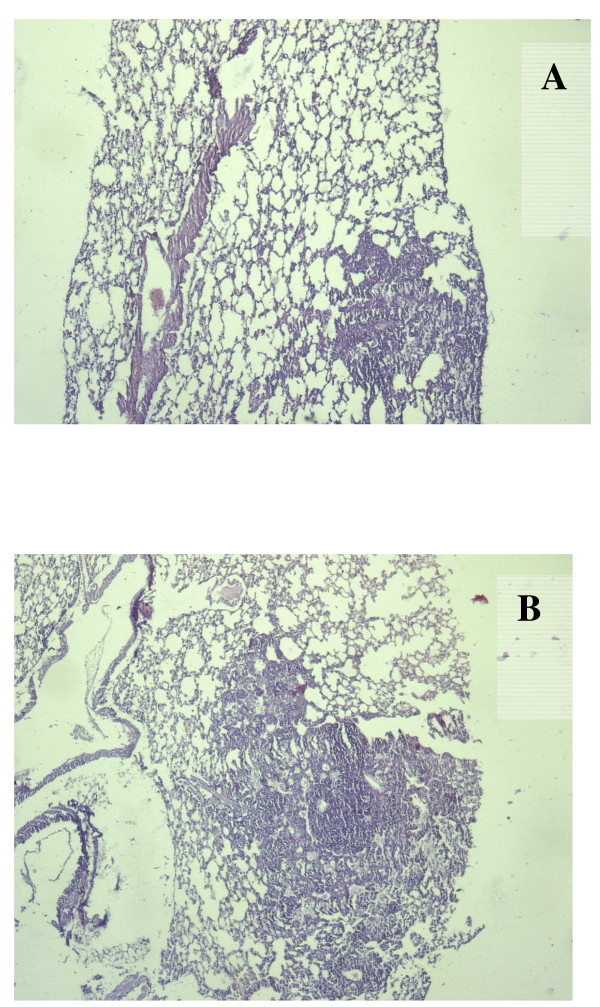
**Histopathology of the lungs of intraperitonealy infected C57Bl/6 mice before (90 days postinfection, A) and after aminoguanidine administration (120 days postinfection, B)**. Lungs were sectioned and stained with hematoxilin and eosin. Magnification, 100×.

Mice were infected intraperitoneally with H37Rv at an initial dose of ca. 10^3 ^CFU per mice and steady-state chronic infection was established in both lungs and spleen as determined by the stable bacillary load in these organs at 50 days post infection (Figure 1). Bacteria appeared first in the spleen and then in lungs similarly with intravenous infection of mice [20]. However, the observed kinetics of MTB multiplication was slower and the final bacillary loads were lower by 1–1.5 orders of magnitude) in comparison with published results for low dose aerogenic infections (see refs.14,22) Administration of AG (a nitric oxide synthase inhibitor which will result in immune suppression [23]) at 90 days postinfection resulted in an increase in CFUs and development of large granulomas in lungs (Figure 2). Histopathologic analysis of the lungs after AG administration revealed areas with decreasing numbers of lymphocytes, increased infiltration of macrophages and overall progression of the granulomatous response in the lungs of infected mice. Furthermore, addition of anti-TNFα antibodies resulted in similar reactivation of bacterial growth in lungs, Figure 1. To prevent the negative side effects of long term immune suppression we used a short-term regimen for administration of these compounds which resulted in a transient increase in CFUs in lungs up to two orders of magnitude above steady-state level. This transition lasted about 2 months, after which the CFUs decreased to the initial level for anti TNFα and to slightly higher level for AG (Figure 1). These data clearly show the applicability of the IP model for modeling of chronic infection in mice with growth and subsequent stabilization of CFUs in organs over time, similarly to aerogenic challenge.

### Assessment of *rpf *deletion mutants in the IP model of chronic infection

**Figure 3:**
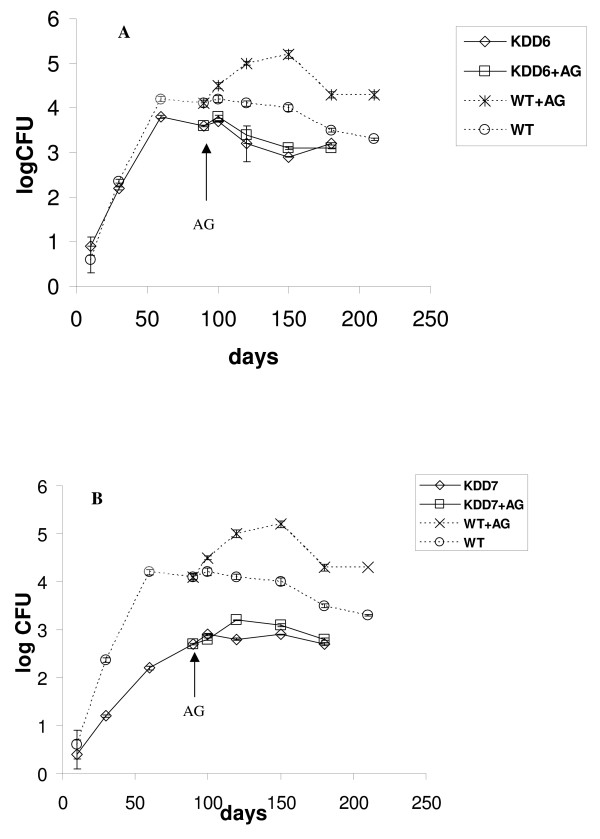
**Growth kinetics of *M. tuberculosis *in lungs after intraperitonel infection of C57Bl/6 mice with double *rpf *deletion mutants**. Infection dose for wild type, KDD6 and KDD7 strains was identical (ca. 10^3 ^CFU per animal). See legend to Figure 1 and Materials and Methods for details. Dotted lines represent the kinetics of infection with the wild type shown on Figure 1 for comparison.

**Figure 4:**
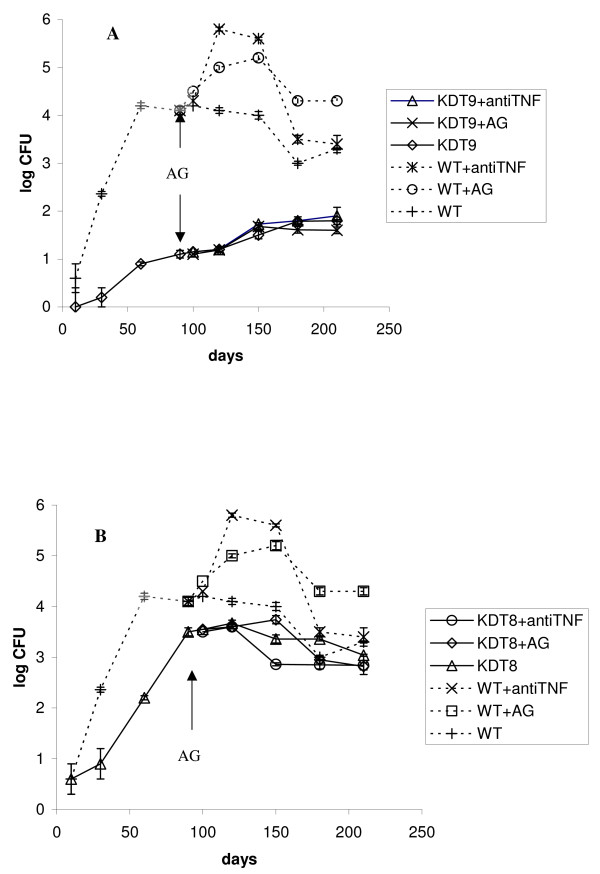
**Growth kinetic of *M. tuberculosis *in lungs after intraperitonel infection of C57Bl/6 mice with triple *rpf *deletion mutants**. Infection dose for wild type, KDT9 and KDT8 strains was identical (ca. 10^3 ^CFU per animal). See legend to Figure 1 and Materials and Methods for details. Dotted lines represent the kinetics of infection with the wild type shown on Figure 1 for comparison.

Using the model described above we examined behavior of MTB strains which were selectively depleted of *rpf *genes in different combinations. We first assessed the double mutants which were deleted for *rpfA *and *rpfC *(KDD7) and deleted for *rpfA *and *rpfB *(KDD6) [20]. The initial IP infection dose was similar to the wild type and both double mutant strains were capable of establishing chronic infection however, the steady-state CFU levels were slightly lower than those observed for the wild type strain, Figure 3A and 3B. The KDD7 mutant appeared to be more attenuated than the KDD6 mutant since the time needed to establish chronic infection was longer for the former. Interestingly an increase in CFUs was observed with KDD7- albeit not as pronounced as for the wild type – during immune suppression and this effect was not seen with KDD6, Figure 3A and 3B. We also assessed triple *rpf *deletion mutants which were depleted of three *rpf *genes, *rpfA *Δ*rpfC *Δ*rpfB *(KDT8) and Δ*rpfA *Δ*rpfC *Δ*rpfD *(KDT9) [20] in our IP model of chronic infection. Both triple *rpf *deletion mutants tested are attenuated for growth in our model and achieved lower CFUs during chronic infection when compared to the wild type, Figure 4A and 4B with KDT9 and KDT8 showing 7 × 10^3 ^and 80 CFUs respectively at the onset of chronic infection. More time is required to establish steady-state levels of CFU in lungs for both triple mutants in comparison with wild type (150 days for KDT9 and 100 days for KDT8 in contrast to 60 days for wild type). In contrast to the wild type the triple mutants did not show a significant increase in CFUs compared to untreated controls upon AG or anti TNFα antibody administration which in this case resulted in the recruitment of mainly lymphocytes to sites of infection in the lungs (not shown). Interestingly, the CFUs for KDT8 decreased during chronic infection and whilst the bacillary load in mice infected with KDT9 showed a modest increase.

## Discussion

In this study we have shown that IP infection does result in chronic TB infection in mouse lungs similar to that observed during low dose aerosol infection. This type of infection is not common as the natural route of TB infection is absorption of bacteria by lung tissues. The kinetics of accumulation of bacteria in the organs after IP administration is slower than that of chronic TB via aerogenic infection (stabilization of CFU numbers at an almost constant level after 60 days vs 20–35 days for aerogenic infection) [14,22]. The advantages of this approach are low level cross contamination between animals and low cost of the experiments (no need a special apparatus for aerogenic infection). Dhillon *et al*. have reported on establishing of chronic TB infection in Balb/c mice with CFU levels of ca 10^4 ^CFU in lungs and in spleens after 4 weeks of IP infection but the kinetic curves were not published [24]. It is interesting that the significant portion of cells recovered from organs in this model [24] were non-culturable on solid medium but capable of growth in liquid media [24]. These observations bear some similarity to dormant MTB cells obtained in vitro [4] and are suggestive of the formation of a population of dormant (latent) bacteria in the IP model. It is interesting to note that a similar population of non-culturable bacteria could also be isolated when MTB was grown in macrophages [25] Evidently, the stable CFUs in organs at 60 days post infection are controlled in an immune dependent fashion as with low dose aerosol infection since immune suppression by AG or anti-TNFα antibodies resulted in significant increase of CFU levels in lungs after 20–30 days of drug administration. Similar results were also observed for chronic infection induced after aerosol challenge [22]. AG is known as an effective inhibitor of nitric oxide synthase (NOS) and induces reactivation of bacillary growth in mice with chronic TB infection [26,27]. As expected AG administration in our model resulted in increased bacillary growth and an increase in the lesion area in the lungs of infected mice (Figure 2). Nitric oxide has been shown to alter the regulation of an endothelial derived mediator of vascular tone, endothelin-1 (ET-1). It was demonstrated that increasing endogenous NO generation through activation of NOS will lead to a decrease in ET-1 [28]. It is possible that AG administration can result in an increase in ET-1 possibly followed by vasoconstriction, pulmonary hypertension and hypoxia. These factors will result in augmentation of lesion area in lungs as found in this study (Figure 2). In the present study the increase in CFU is transient in contrast to published results [22,26]) as AG was given to mice for a short period only (10 days). This approach makes it possible to observe the direct response of bacterial multiplication to inhibition of NOS without interference by the negative site effects caused by long-term administration of AG. A similar reactivation effect was observed after administration of anti-TNFα antibodies. It has been previously shown that anti-TNFα antibodies were able to induce reactivation of TB in chronically infected mice and produced significant histopathological deterioration in organs [27]. This effect of inactivation of TNFα is probably due to different mechanisms including modulation of inflammatory state of the lungs, attenuation of the expression of NOS2 in the lungs and altered expression of other cytokines and chemokines [27,29]. The increase in CFUs after anti-TNFα treatment in the present study was similar to data reported in previous experiments with chronically infected mice (ca. 1.5 log after administration) [27]. Again, due to the short regimen of antibody administration, the increase in CFUs is transient.

Generally, the IP infection model reveals features similar to the more commonly used low dose aerogenic infection model, such as the establishment a steady-state level of CFUs in organs that does not change significantly over time. Given that clinical latency in humans is characterized by low bacillary loads, the IP route of infection may in fact model TB latency even better than aerogenic infection as the steady-state bacterial load in this study was lower than for aerogenic infection for the same strain in our studies (unpublished results and see also [14,15,30]).

In a previous study, the behavior of MTB strains that were deleted for either one or three *rpf *genes were investigated *in vitro *and *in vivo *and we found that these mutants did not show significant growth attenuation *in vitro *although the triple mutants were significantly and differentially attenuated in B6 mice [20]. Similarly, double mutants used in this study grew as well as the wild type in Sauton's medium either in broth culture or on plates (data not shown). These strains have also been checked for their ability to resuscitate from a "non-culturable" state after prolonged incubation in stationary phase under anaerobic conditions [20]. Strains deleted for one *rpf *gene only showed no defects in their ability to resuscitate however, the triple mutants were significantly defective for resuscitation in this model. This defect for the triple mutants could be restored by addition of culture filtrate (taken from log phase MTB cells) to the resuscitation medium [B. Kana *et al*., in preparation] indicating that the inability of bacteria to spontaneously resuscitate was not only due to poor viability of the mutant strains in this model. We tested KDD6 for its ability to recover from a non-culturable state *in vitro *and we found no defects (data not shown).

In this study we show that one double (KDD7) and both triple *rpf *deletion mutants reveal significant attenuation in a chronic *in vivo *model after IP infection. Moreover, deletion of three *rpf *genes resulted in complete arrest of cell multiplication after immune suppression in the case of KDT8. In our model KDD7 seems to be more attenuated than KDT8 (which was derived from KDD7) at 90 days (3.5 log CFUs for KDT8 vs 2.9 log for KDD7) (Figures 3B and 4B). However, this difference is reduced as the experiment progresses and could also be associated with some natural variation in mice sensitivity in different groups. Deletion of the *rpfA*, *rpfC *and *rpfD *genes (KDT9) resulted in greater attenuation of MTB in the IP model than deletion of the *rpfA*, *rpfC *and *rpfB *genes (KDT8) (Figs 4A,B), in contrast to previously published data that indicated KDT8 was more attenuated for growth in B6 mice than KDT9 [20]. We speculate that this discrepancy could be related to the different models used in the published experiment (intravenous, acute infection, B6 mice) compared to the current study. It is possible that the Rpf proteins could be differentially important for MTB proliferation depending on the route of bacillary dissemination. We are currently attempting to genetically complement the multiple *rpf *deletion strains and further pair wise experiments with complemented derivatives should confirm unequivocally the role of Rpfs in pathogenesis. However, our data are reflective of the notion that Rpf proteins may have a significant physiological role under conditions of bacterial stress in vivo. Indeed, actively grown mycobacteria were not sensitive to Rpf [18] whilst aged cultures or "non-culturable" cells were sensitive to Rpf addition in form of exogenously administrated protein or the plasmid coding for Rpf synthesis [18,31]. We also found that bacterial growth from a small inoculum in non-optimum medium was sensitive to Rpf administration [18]. Chronic infection (low MTB initial dose) could be considered as less permissive for mycobacterial multiplication in comparison with the acute infection phase and therefore, the role of Rpf proteins in the development of chronic TB infection and reactivation become more prominent and it is clear that different Rpfs contribute differently to the observed effect. It seems that RpfC is important for bacterial dissemination in the IP model in double knockouts while RpfD plays a more significant role in triple mutant combinations. At the same time RpfB is important for AG induced reactivation for the double mutants which is in accordance with the observations of Tufariello *et al. *for a single *rpfB *mutant [22].

The observed behavior of the different *rpf *mutants *in vivo *elucidates a sophisticated interplay between different Rpf proteins during cell growth and cell recovery after dormancy. Indeed, being almost redundant in single mutants, Rpf proteins exhibited less redundancy in double and triple mutants. We speculate that the different Rpfs perform similar functions in the cell (e.g. cell wall processing [32]) but at different loci of the target or with different efficiencies. This is supported by the fact that all Rpf molecules from MTB show differences in their variable domain which is probably important for cell wall binding [30]. In multiple mutants it is likely, that some of the remaining molecules could substitute for the deleted Rpf better than others and the probability of such successful substitution decreases in the order of single-double-triple mutants. Alternatively, expression of a particular *rpf *gene could influence (directly or indirectly) the expression of the others. Future studies should elucidate these possibilities. Despite the fact that the mechanisms of Rpf activity are not fully understood it is clear that these proteins represent interesting targets for the design of chemical compounds against development of chronic\ latent TB infection. If dormant MTB cells sustain some metabolic activity, such anti-Rpf compounds could not only stop reactivation disease but eventually kill latent organisms.

## Conclusion

1. IP infection results in chronic TB infection in mouse lungs similar to that observed during low dose aerosol infection.

2. Rpf proteins may have a significant physiological role for development of chronic TB infection and its reactivation *in vivo*.

## Competing interests

The author(s) declare that they have no competing interests.

## Authors' contributions

BK and KD carried out the molecular genetic studies, made KO mutants, BK participated in the manuscript drafting. SB and VP carried out IP mouse model. EG participated in histopathology studies. ASK conceived of the study, and participated in its design and coordination and helped to draft the manuscript. All authors read and approved the final manuscript.

## Pre-publication history

The pre-publication history for this paper can be accessed here:


